# Pharmacokinetics and Pharmacodynamics of (*S*)-Ketoprofen Co-Administered with Caffeine: A Preclinical Study in Arthritic Rats

**DOI:** 10.3390/pharmaceutics10010020

**Published:** 2018-01-26

**Authors:** Raúl Medina-López, Nancy Vara-Gama, Olivia Soria-Arteche, Luis A. Moreno-Rocha, Francisco J. López-Muñoz

**Affiliations:** 1 Departamento Sistemas Biologicos Universidad Autonoma Metropolitana-Xochimilco, Calz. del Hueso 1100, Col. Villa Quietud, Mexico City 04960, Mexico; inukiniro@yahoo.com.mx (N.V.-G.); soriao@correo.xoc.uam.mx (O.S.-A.); alfonso.moreno72@gmail.com (L.A.M.-R.); 2 Laboratorio No. 7 “Dolor y Analgesia” del Departamento de Farmacobiologia, Cinvestav-Sede Sur, Calz. de los Tenorios No. 235, Col. Granjas Coapa, Mexico City 14330, Mexico; flopezm2@gmail.com

**Keywords:** (*S*)-ketoprofen, caffeine, pharmacokinetics, pharmacodynamics, PIFIR model

## Abstract

The purpose of the present study was to determine whether caffeine modifies the pharmacokinetics and pharmacodynamics of (*S*)-ketoprofen following oral administration in a gout-type pain model. 3.2 mg/kg of (*S*)-ketoprofen alone and combined with 17.8 mg/kg of caffeine were administered to Wistar rats and plasma levels were determined between 0.5 and 24.0 h. Additionally, antinociception was evaluated based on the protocol of the PIFIR (pain-induced functional impairment in the rat) model before blood sampling between 0.5 and 4.0 h. Significant differences in C_max_, AUC_0-24_, and AUC_0-∞_ values were observed with caffeine administration (*p* < 0.05). Also, significant differences in E_max_, T_max_, and AUC_0-4_ values were determined when comparing the treatments with and without caffeine (*p* < 0.05). By relating the pharmacokinetic and pharmacodynamic data, a counter-clockwise hysteresis loop was observed regardless of the administration of caffeine. When the relationship between AUCe and AUCp was fitted to the sigmoidal *E*_max_ model, a satisfactory correlation was found (R^2^ > 0.99) as well as significant differences in *E*_max_ and *EC_50_* values (*p* < 0.05). With caffeine, *E*_max_ and *EC_50_* values changed by 489.5% and 695.4%, respectively. The combination studied represents a convenient alternative for the treatment of pain when considering the advantages offered by using drugs with different mechanisms of action.

## 1. Introduction

The adequate treatment of pain originated by inflammatory processes is sought worldwide. The primary objective of pharmacokinetic/pharmacodynamic modeling is to identify key properties of a drug in vivo, which allows the characterization and prediction of the time course of drug effects under physiological and pathological conditions [1]. Some of the potential benefits of modeling in the preclinical phase are the prediction of clinical potency, provision of guidance for optimal sampling, and prediction of oral bioavailability and hepatic clearance, among others [2]. The process of modeling data from clinical drug trials involves fitting mathematical equations to concentration, time, and/or effect data in an attempt to impart some order to the observations [3].

The pharmacokinetic and pharmacodynamic performance of some analgesic drugs has been previously published [4]. Determination of plasma levels and an arthritic-pain animal model have been specifically used for the evaluation of paracetamol [5], ketorolac [6], diclofenac [7], and tolmetin [8]. The combination of analgesic drugs and caffeine can produce better analgesic effects than the administration of drugs alone. To demonstrate this, pharmacokinetic/pharmacodynamic performance with the concomitant administration of caffeine has been studied for paracetamol [9], aspirin [10], and naproxen [11]. Additionally, the analgesic effects of several doses of caffeine combined with some doses of racemic ketoprofen [12], as well as with some doses of racemic ibuprofen [13], have been previously reported. Results were compared with the administration of drugs alone. On the other hand, the antinociceptive effect of the enantiomer, which has shown biological activity in arthritic-pain animal models, has been studied for *S*(+)-ketoprofen [14] and *S*(+)-flurbiprofen [15]. All these preclinical studies provide information needed to characterize and predict the time course of some non-steroidal anti-inflammatory drugs (NSAIDs).

Ketoprofen (2-(3-benzoylphenyl) propionic acid) is an NSAID of the series of arylcarboxylic acid derivatives, with anti-inflammatory, analgesic, and antipyretic properties. It inhibits cyclooxygenase, which catalyzes the formation of prostaglandin precursors from arachidonic acid [16]. Its efficacy has been demonstrated in the treatment of rheumatoid arthritis, osteoarthritis, ankylosing spondylitis, gout (acute episodes), pain associated with inflammation, dental pain, trauma, and postoperative pain [12]. Ketoprofen has two enantiomers: (*R*)- and (*S*)-ketoprofen, and it is well documented that it is the latter that produces the analgesic effects [17]. The (*S*)-isomer inhibits in vitro prostaglandin synthesis [18,19]. The analgesic effect of (*S*)-ketoprofen has been demonstrated in animal models [20,21] and in humans [22].

Caffeine is an alkaloid named “3,7-dihydro-1,3,7-trimethyl-1*H*-purine-2,6-dione”. It is a psychoactive and central nervous system stimulant of the methylxanthine class [23]. It has been used as an adjuvant in a wide variety of pain stages, both acute and chronic, in combination with NSAIDs and with non-opioid analgesic compounds, such as acetylsalicylic acid and acetaminophen. Pharmaceutical dosage forms with these combinations have already been on the market for many years. The antinociceptive effects resulting from combining caffeine with some analgesic drugs have been examined [12,24,25]. The results are in some cases contradictory, mostly due to the use of different species, doses, routes of administration, and antinociception models. In summary, these controversies [13,26,27], especially in the clinical tests, are due to differences in the protocols used, as well as inter-individual variations that are quite high [28]. A controlled experimental study would be very useful to clarify the discrepancies, although it is not easy to find changes related to the efficacy of the drug, mainly due to certain limitations in the clinical trials (a relatively small number of subjects, valuations through subjective scales, and differences in dosing regimens).

The experimental animal model, named “pain-induced functional impairment in the rat” or PIFIR model, has been previously described [29]. In this model, nociception and antinociception take place in a similar way to that found in arthritic gout-type pain situations. This experimental procedure helps to determine potency, efficacy, and duration of the antinociceptive effect produced by a drug administered alone or in combination with another drug. It has been used for some works mentioned above. The PIFIR model is an adequate tool to investigate the pharmacokinetic and pharmacodynamic relationships in preclinical studies, and to our knowledge the (*S*)-ketoprofen–caffeine pharmacokinetic/pharmacodynamic interaction has not yet been characterized. Therefore, the purpose of the present study was to further examine the relationship between (*S*)-ketoprofen and caffeine by determining whether the latter alters both the pharmacokinetics and analgesic effect of this active enantiomer following oral administration to experimental animals in a gout-type pain model.

## 2. Materials and Methods

### 2.1. Drugs

Analytical standards of (*S*)-ketoprofen, caffeine, and uric acid were obtained from Sigma Chemical Co. (St. Louis, MO, USA) and suspended in mineral oil. Either (*S*)-ketoprofen alone or in combination with caffeine was dissolved in an alcoholic solution (10% ethanol). Uric acid was suspended in mineral oil (30%). Drug solutions were freshly prepared and administered at a volume of 2 mL/kg of body weight.

### 2.2. Animals

The studies were carried out on male Wistar rats (Crl (WI) fBR) with an approximate weight between 180 and 220 g. All the experiments were carried out following the recommendations of the Research and Ethics Committee of the International Association for the Study of Pain, the Guidelines and Ethical Standards for the Investigation of Experimental Pain in Animals, and according to an experimental protocol (Protocol No. 16, 29 January 2014) that meets the requirements established in the official Mexican Standard (NOM-062-ZOO-1999), and conformed to the Internationally Accepted Principles of Use and Care of Laboratory Animals [30]. The animals were kept in a room at constant temperature (22 ± 2 °C), with day/night cycles of 12 h. The rats received standard feed (Purina Laboratory Rodent Diet 5001, Pet Food, Richmond, IN, USA). Approximately twelve hours before the experiment, they were fasted and kept with water *ad lubitum*. The number of experimental animals was kept to a minimum. The animals were provided by the Laboratory Animal Production and Experimentation Unit (UPEAL) of the Universidad Autonoma Metropolitana-Xochimilco. In this study, groups of rats were used for the administration of (*S*)-ketoprofen, either alone or in combination with caffeine. An alcoholic solution and uric acid suspension were used as controls. At the end of the study, the rats were sacrificed inside an isoflurane chamber to avoid unnecessary suffering.

### 2.3. Pharmacokinetics of (S)-Ketoprofen

#### 2.3.1. Blood Sampling

On the day of the study, the rats were cannulated approximately 30 min before uric acid injection and 3.0 h before the drugs were administered. The animals were anesthetized and the caudal artery was cannulated with a PE-10 cannula (Clay Adams, Parsippany, NJ, USA) connected to a PE-50 cannula. The cannula was kept patent with heparinized saline solution and stopped with a needle. The rats were allowed to recover from anesthesia and 3.2 mg/kg of (*S*)-ketoprofen alone or in combination with 17.8 mg/kg of caffeine was orally administered. The choice of these doses will be discussed later. Blood samples were withdrawn from the caudal artery at 0 h (before the administration of the drug) and at 0.5, 1.0, 1.5, 2.0, 2.5, 3.0, 3.5, 4.0, 6.0, 8.0, and 24.0 h after drug(s) administration. The cannula was withdrawn and the animals were sacrificed after the last sample was taken. Blood samples were transferred to heparinized polypropylene tubes. Plasma was separated by centrifugation at 3500 rpm for 10 min at 4 °C and stored at −20 °C until further analysis. The total volume of blood taken from each animal did not exceed 2.4 mL.

#### 2.3.2. Sample Preparation

Extraction of (*S*)-ketoprofen from plasma samples was conducted using a solid-phase extraction (SPE) technique previously developed in our laboratory [31]. Briefly, the cartridges (Strata-X 33 µm polymeric reversed phase cartridge, Phenomenex, Torrance, CA, USA) with the aid of a vacuum device (Vac-Elut, Speed Mate 10, Applied Separations Inc., Allentown, PA, USA) were preconditioned by flushing with 2 mL of methanol and 1 mL of high-performance liquid chromatography (HPLC) water. Separately, 50 μL of plasma sample plus 100 μL of an 85% phosphoric acid:water mixture (1:10) and 10 μL of internal standard solution (diclofenac (Sigma Chemical Co., St. Louis, MO, USA) at 100 μg/mL) were vortex mixed. Then, samples were loaded into the cartridge and allowed to stand for 5 min, washed with 0.6 mL of a water:methanol mixture (95:5, *v*/*v*) and dried under vacuum. The (*S*)-ketoprofen was eluted with 1 mL of an acetonitrile:methanol mixture (50:50, *v*/*v*) at a flow rate of 1 mL/min. The eluate was evaporated to dryness in a water bath at 37.0 ± 0.5 °C under a gentle stream of nitrogen (Praxair Mexico, Mexico City, Mexico). The residue was reconstituted in 50 μL of mobile phase and 30 μL was injected onto the HPLC system. Acetonitrile, methanol, and 85% phosphoric acid HPLC-grade were used (J.T.Baker, Xalostoc, Mexico). Water HPLC-grade (18 Ω) was obtained by purifying distilled water in a Milli-Q filtration system (Millipore, Bedford, MA, USA).

#### 2.3.3. Chromatographic Conditions

(*S*)-ketoprofen plasma concentrations were determined by HPLC analysis with ultraviolet (UV) detection [31]. The chromatographic system consisted of a Perkin Elmer Series 200 HPLC (Norwalk, CT, USA) equipped with a binary pump, a manual injector with a 20 μL loop, and a variable wavelength UV 785 model detector (Applied Biosystems, Foster, CA, USA). PE Nelson-Turbochrom software was used for data acquisition and processing. Separation was performed on a (*S*,*S*)-Whelk-0 1 stainless steel column, with 5 μm particle size and 250 × 4.6 mm (Regis Technologies Inc., Morton Grove, IL, USA). A Phenomenex security guard column packed with CN (cyanopropyl silica) material (4.0 × 0.3 mm, Torrance, CA, USA) was used before the analytical column. The mobile phase consisted of a mixture of hexane–ethanol–acetic acid (93:7:0.5, *v*/*v*/*v*), degassed before use, and the flow rate was 1.5 mL/min. Hexane, ethanol, and acetic acid HPLC-grade were used (J.T.Baker, Xalosctoc, Mexico). Detection wavelength was set at 254 nm. All analyses were carried out at room temperature (25 °C). This method has proven to be linear (R^2^ ≥ 0.999), precise (relative standard deviation < 15%), and accurate (relative error ±12%) within a range of 0.78–12.5 μg/mL.

### 2.4. Pharmacodynamics of (S)-Ketoprofen

#### 2.4.1. Arthritis Induction

Antinociception was assessed using the PIFIR model [29]. Briefly, the animals were anesthetized inside an isoflurane chamber. Nociceptive dysfunction was induced on the patella of one of the animal’s hind legs, by intra-articular injection of 50 μL of 30% *v*/*v* uric acid suspension. Immediately, an electrode was attached to the plantar region of both hind limbs. The animals were allowed to recover from the anesthetic and placed on a 30 cm diameter stainless steel cylinder. The cylinder was rotated at a speed of 4 rpm forcing the animals to walk. The variable to be measured was the contact time of each electrode on the cylinder. When each electrode placed on the legs of the animal touches the metal surface of the cylinder, the circuit closes and the time that the circuit remains closed was recorded.

#### 2.4.2. Behavioral Assessment

The percentage ratio between the contact time of the administered foot with respect to the non-administered one was expressed as percentage of functionality index (FI%). The cylinder was operated for 2 min with intervals of 15 or 30 min of rest until completion of the test. In all experiments, the drug alone as well as the corresponding combination was dissolved in alcoholic solution and were orally administered. Then, 2.5 h after the injection of uric acid, time was considered as the initial time or “zero” for the measurement of antinociceptive effects and the (*S*)-ketoprofen treatment at 3.2 mg/kg alone or combined with caffeine, was administered. Data for the corresponding FI% were recorded immediately before the blood sampling at 0.5, 1.0, 1.5, 2.0, 2.5, 3.0, 3.5, and 4.0 h. All the experiments were carried out on a fixed schedule running from 7:00 to 14:00 h. After the experiments, 24 h later, the animals were sacrificed after the last blood sample. In this work, the induction of nociception in animals was inevitable; however, it was avoided as far as possible to cause unnecessary suffering in the animals.

### 2.5. Pharmacokinetic and Pharmacodynamic Data Analysis

(*S*)-ketoprofen plasma concentration–time curves were constructed and data were evaluated by non-compartmental analysis. From the time courses obtained, the following pharmacokinetic parameters were determined: maximum plasma concentration (C_max_), time to achieve this value (T_max_), area under the curve from zero time until 24.0 h after drug(s) administration (AUC_0-24_), area under the curve from zero time to infinity (AUC_0-__∞_), rate constant of the terminal phase of elimination (λ_z_), half-life of elimination (t_½_), and mean residence time (MRT). Non-compartmental analysis was performed using the Excel add-on PKSolver [32]. Treatment effect on pharmacokinetic parameters was tested using unpaired Student’s *t*-test after logarithmic transformation. Differences were considered statistically significant if *p* < 0.05. Antinociceptive temporal courses for (*S*)-ketoprofen alone and combined with caffeine were plotted for each treatment. Mean E_max_ and T_max_ were calculated directly from the observed data. Area under the effect–time curve (AUC_0-4_) values were calculated by the trapezoidal rule [33]. Treatment effect on E_max_, T_max_, and AUC_0-4_ was tested using unpaired Student’s *t*-test. Differences were considered statistically significant if *p* < 0.05. In order to investigate the relationship between the antinociceptive effects observed and (*S*)-ketoprofen plasma concentrations under different experimental conditions, mean FI% was plotted against mean plasma concentrations at each sampling time during the first 4.0 h. If the resulting curve exhibited a counter-clockwise hysteresis loop, then a distribution delay between the systemic drug concentration and the time to reach the effect site was suggested. Finally, as a previously reported technique [34], the cumulative area under the effect–time curve (AUCe %h) vs. the cumulative area under the plasma concentration–time curve (AUCp μg·h/mL) for (*S*)-ketoprofen alone and combined with caffeine was plotted and fitted to the sigmoidal *E*_max_ model according to the Hill’s equation:

(1) $$E=\frac{E_{max}C^{\gamma }}{EC_{50}^{\gamma }+C^{\gamma }}$$

where *E* is the cumulative observed overall effect (cumulative AUCe), *E*_max_ is the theoretical maximal cumulative AUCe value that can be attained, *C* is the cumulative observed overall plasma level (cumulative AUCp), *EC_50_* is the cumulative AUCp that induces a cumulative effect equivalent to 50% of *E*_max_, and *γ* is the response factor (Hill’s coefficient). The fit model for the relationship between AUCp and AUCe was carried out using the Excel add-on PKSolver [32].

## 3. Results

### 3.1. Pharmacokinetics of (S)-Ketoprofen

**Figure 1:**
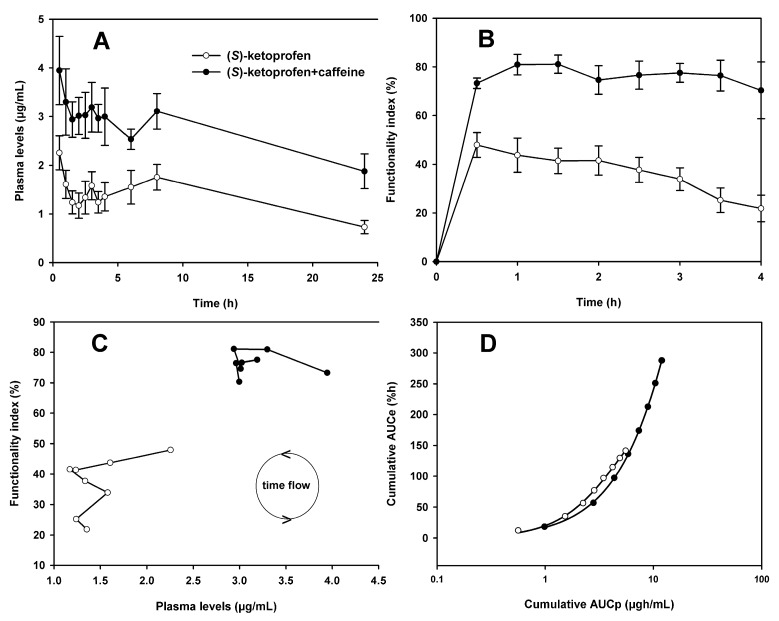
Results obtained after p.o. administration of (*S*)-ketoprofen alone (3.2 mg/kg) and in combination with caffeine (17.8 mg/kg) to male Wistar rats (Mean ± SEM, *n* = 6/group). (**A**) Plasma concentration–time curves. (**B**) Antinociceptive effect–time curves. (**C**) Antinociceptive effect vs. plasma concentrations. (**D**) Cumulative AUCe as a function of cumulative AUCp, data adjusted to sigmoidal *E*_max_ model.

Mean plasma concentrations of (*S*)-ketoprofen alone (3.2 mg/kg) and in combination with caffeine (17.8 mg/kg), plotted as a function of time, are shown in Figure 1A. At all sampling times, caffeine administration increased plasma levels of (*S*)-ketoprofen. Pharmacokinetic parameters calculated using non-compartmental analysis are shown in Table 1. Significant differences in C_max_, AUC_0-24_, and AUC_0-∞_ values were observed when comparing the (*S*)-ketoprofen treatment with that of the (*S*)-ketoprofen + caffeine combination (*p* < 0.05). The values of these parameters increased by 90.1%, 82.7%, and 57.6%, respectively. However, no significant differences in λ_z_, t_½_, and MRT values were found (*p* > 0.05).

### 3.2. Pharmacodynamics of (S)-Ketoprofen

The intra-articular injection of uric acid resulted in the complete dysfunction of the right paw in a period of approximately 2.5 h, which corresponded to FI% = 0. At that time (zero time), drug(s) were administered. At the doses of (*S*)-ketoprofen and caffeine used, no adverse effects that could interfere with the course of the study or the recording of the data (FI%) were observed. The cumulative antinociceptive effect during the observation period (4.0 h) was determined as AUC_0-4_ in order to analyze the whole antinociception effect elicited by (*S*)-ketoprofen alone or by its combination with caffeine. The antinociceptive effects as a function of time elicited by (*S*)-ketoprofen and the combination of (*S*)-ketoprofen + caffeine are shown in Figure 1B. The pharmacodynamic parameters obtained after p.o. administration of (*S*)-ketoprofen alone and in combination with caffeine are shown in Table 1. Significant differences in E_max_, T_max_, and AUC_0-4_ values were observed when comparing the treatments with and without caffeine (*p* < 0.05). The values of these parameters increased by 69.1%, 140.9%, and 103.6%, respectively. When caffeine was administered, the (*S*)-ketoprofen time-course curve reached FI values up to 70%, while at the end of the experiment without caffeine administration FI values below 22% were found.

### 3.3. Pharmacokinetic and Pharmacodynamic Data Analysis

FI% data as a function of (*S*)-ketoprofen plasma concentrations are presented in Figure 1C, which shows a counter-clockwise hysteresis loop with and without caffeine administration. A simple way to achieve the hysteresis collapse is to match AUCe vs. AUCp; the resulting plots of (*S*)-ketoprofen alone and in combination with caffeine are shown in Figure 1D. When the relationship between AUCe and AUCp was adjusted to a sigmoidal *E*_max_ model, for both treatments, a satisfactory correlation (R^2^ > 0.99) could be observed. Results of these fits are shown in Table 1. Upon comparing the treatment with and without caffeine, significant differences in *E*_max_, and *EC_50_* values were found (*p* < 0.05). When caffeine was administered, *E*_max_ and *EC_50_* values changed by 489.5% and 695.4%, respectively.

## 4. Discussion

The enantiospecific pharmacokinetics of ketoprofen have been studied in several animal species; in some species, one enantiomer prevails over the other, and in other species both enantiomers appear similar. In humans, rats, and mice, absorption is not stereoselective [35]. The pharmacokinetics of (*S*)-ketoprofen alone and in combination with caffeine have been previously determined in other studies [31]. After administration of a single dose of 3.2 mg/kg of (*S*)-ketoprofen or 3.2 mg/kg of (*S*)-ketoprofen + 17.8 mg/kg of caffeine to Wistar rats, significant differences in C_max_ and AUC_0-∞_ of (*S*)-ketoprofen were found (*p* < 0.05). In our work, where additionally the antinociceptive effect was simultaneously determined, similar results were found since the pharmacokinetic parameters C_max_, AUC_0-24_, and AUC_0-∞_ showed significant differences. Caffeine produced higher plasma levels of (*S*)-ketoprofen when it was administered (Figure 1A), but no significant changes in other important pharmacokinetic parameters, such as λ_z_, t_½_, and MRT, were found. Some component or mechanism caused (*S*)-ketoprofen plasma concentrations to increase, but not in the way that this compound was metabolized or eliminated. Considering the temporal courses of plasma concentrations of (*S*)-ketoprofen in Figure 1A, a second peak of drug is observed, after administration of (*S*)-ketoprofen alone and in combination with caffeine, that could be due to enterohepatic recycling. Ketoprofen stereoselective enterohepatic circulation has been reported in rats [36] and double (*S*)-ketoprofen peaks have been found in pigs [37]. For a maximum anti-inflammatory effect in arthritic rats, serum concentrations of 0.2–0.4 μg/mL of (*S*)-ketoprofen were found [38]. These values were different from those found in our study due to differences in the protocols used. It is noteworthy that after oral administration of (*S*)-ketoprofen alone or in combination with caffeine, plasma concentrations of the inactive (*R*)-ketoprofen enantiomer were not found. This is in agreement with other reports in which plasma levels of (*R*)-ketoprofen were not found after intravenous [39] and oral [14] administration of (*S*)-ketoprofen to rats. In this sense, ketoprofen undergoes unidirectional chiral inversion from the (*R*)- to (*S*)-enantiomer. The extent of inversion varies considerably among species [35]. The specific pharmacokinetic mechanism by which caffeine is capable to increase plasma levels of (*S*)-ketoprofen is not entirely clear. Caffeine may increase the absorption of (*S*)-ketoprofen because caffeine decreases gastric pH, reduces gastric emptying, and increases blood flow in the microcirculation of the gastric mucosa. In addition, caffeine decreases liver blood flow, which may reduce metabolic clearance [12]. It has been reported that the systemic administration of caffeine to rats and mice at doses lower than 10–50 mg/kg generally has no intrinsic antinociceptive effects [12]. The accepted mechanism of action for caffeine is that it can block adenosine receptors that have a certain affinity for methylxanthines [40]. Adenosine produces its effects in a variety of systems and often turn out to be opposite to those produced by caffeine. Blockade of adenosine receptors by methylxanthines has been directly implicated in the pharmacological effects of caffeine in humans [41].

The pharmacodynamics of (*S*)-ketoprofen alone and in combination with caffeine were previously studied [42]. Several doses of (*S*)-ketoprofen (0.1–31.6 mg/kg) were administered alone and in combination with 17.8 mg/kg of caffeine, and the antinociceptive effects were evaluated in an arthritic-pain animal model. In the study, no plasma levels of (*S*)-ketoprofen were monitored. According to the experiments, the maximum potentiation of antinociceptive effects was found with the co-administration of 3.2 mg/kg of (*S*)-ketoprofen + 17.8 mg/kg of caffeine (*p* < 0.001). Therefore, this combination of drugs was used in our study to monitor, in the same animals, the (*S*)-ketoprofen pharmacokinetic/pharmacodynamic relationship. The results referring to the administration of the combination of (*S*)-ketoprofen and caffeine found in this work confirmed the potentiation of the antinociceptive effects caused by the drugs administered alone (*p* < 0.05), Figure 1B. The mechanisms involved in the analgesic activity of ketoprofen and other NSAIDs are located at several sites throughout the nervous system. It has been suggested that it is due to a peripheral mechanism [43] and this was confirmed in other investigations [16]. In addition to this peripheral mechanism, which is well known, a central component has been found [44,45]. NSAIDs, such as ketoprofen, are able to cross the blood–brain barrier [46] and reach the brain structures that are involved in the regulation of pain sensation [12].

The hysteresis cycles suggest that the relationship between drug concentration and the measured effect is not a simple direct relationship, but rather an inherent delay can be observed as a result of different pharmacokinetic and pharmacodynamic mechanisms including delay in distribution, feedback regulation, tolerance, uptake at the active site, agonistic or antagonistic active metabolites, time-dependent protein binding, slow receptor kinetics, delayed or modified activity, and the use of racemic drugs [47]. In the present study, when the antinociceptive effect of (*S*)-ketoprofen was plotted against plasma concentrations, a counter-clockwise hysteresis loop was displayed. This result was observed with both treatments but when caffeine was administered, the counter-clockwise hysteresis loop was displaced to the right and above of the original position (Figure 1C) meaning that caffeine does not modify this kind of relationship at higher (*S*)-ketoprofen plasma levels and antinociceptive effects. Counter-clockwise hysteresis cycles can occur due to a delay in the distribution between the systemic drug concentration and the time that it takes to reach the effect site [47]. The drugs or the combination of drugs often act through a mechanism of action that is indirect and the pharmacological effect takes longer to become evident, so the response is governed by the stimulation or inhibition of the factors that modulate the answer [48].

When cumulative AUCe was plotted as a function of cumulative AUCp, a sigmoidal relationship was found. This association was observed with and without caffeine administration. The relationship between the concentration of a drug and its effect is often adjusted to a sigmoidal *E*_max_ model (Equation (1)). This mathematical relationship is based on the receptor theory that defines the concentration and effect relationship with two parameters: *E*_max_ and *EC_50_*. This relationship allows for shape differences, where *γ* is the number of molecules that combines with each receptor molecule that affects the shape of the curve [47]. In our work, caffeine does not change this association (R_obs-pre_ > 0.99 in both treatments) but allows for a significant increase in *E*_max_ and *EC_50_* values. It is important to note that even though (*S*)-ketoprofen plasma concentrations markedly increased in the presence of caffeine, the response factor (*γ*) obtained in the sigmoidal *E*_max_ fitting remained unchanged, just like λ_z_, t_½_, and MRT, which may indicate that the effect produced by the combination of (*S*)-ketoprofen + caffeine strongly depends on pharmacodynamic interactions. It is known that caffeine in combination with some NSAIDs can inhibit nociception by blocking adenosine receptors (A_1_ and A_2_) and this may be secondary to the activation of descending serotonergic pathways and the subsequent release of adenosine within the spinal cord [49].

The linkage of pharmacokinetics and pharmacodynamics is taking on great importance because of the necessity to understand the concentration–time profiles of drugs and acquire the ability to determine dosing regimens that will achieve the necessary concentrations for optimal efficacy [47]. Dose–response curves are difficult to perform in clinical situations. The mathematical models themselves are often empiric and, while seeming to describe the effect–concentration relationship adequately, they may not be grounded in physiological reality [3]. In this way, experimental studies seem to be the appropriate alternative for the characterization of caffeine as an analgesic adjuvant [50]. The results of the present study with (*S*)-ketoprofen highlight a direct correlation between the magnitude of the antinociceptive effect and its plasma levels. To our knowledge, this is the first work that not only evaluates the antinociceptive effect of some NSAIDs using the PIFIR model, but it also shows that caffeine significantly increases its plasma levels. Previously, when caffeine was co-administered with this kind of drugs, only a significant increase in the antinociceptive effect was reported [9,10,11].

## 5. Conclusions

The combination of (*S*)-ketoprofen and caffeine (3.2 + 17.8 mg/kg), after p.o. administration, shows important benefits compared to the drugs given alone because it improves the antinociceptive efficacy. The simultaneous administration of caffeine does not modify the pharmacokinetics of (*S*)-ketoprofen, so there are no pharmacokinetic interactions between caffeine and the disposition of (*S*)-ketoprofen. The potentiation of the antinociceptive effect exerted by the combination of these drugs could be explained by other pharmacodynamic interactions involving central and peripheral mechanisms of action; nevertheless these particular mechanisms have not yet been totally explained. However, it should be noted that the pharmacological response is not only due to the rate at which it binds to its receptor, but may also be related to a series of pharmacodynamic mechanisms that can alter or cause a delay in its pharmacological activity. The combination studied represents a convenient alternative for the treatment of pain when considering the advantages offered by using drugs with different mechanisms of action.

## Figures and Tables

**Table 1:** (*S*)-ketoprofen parameters. Mean ± SEM, *n* = 6. * *p* < 0.05.

Pharmacokinetics	(*S*)-Ketoprofen	(*S*)-Ketoprofen + Caffeine
C_max_ (μg/mL)	2.73 ± 0.27	5.19 ± 0.59 *
T_max_ (h)	0.50 ± 0.00	0.50 ± 0.00
AUC_0-24_ (μg·h/mL)	34.49 ± 6.11	63.03 ± 7.66 *
AUC_0-__∞_ (μg·h/mL)	71.17 ± 18.58	112.17 ± 24.78 *
λ_z_ (h^−1^)	0.0389 ± 0.01	0.0404 ± 0.01
t_½_ (h)	26.94 ± 9.22	18.45 ± 3.45
MRT (h)	38.38 ± 12.72	26.66 ± 5.71
**Pharmacodynamics**		
E_max_ (%)	47.93 ± 5.07	81.09 ± 3.74 *
T_max_ (h)	0.83 ± 0.25	2.0 ± 0.52 *
AUC_0-4_ (%h)	141.35 ± 18.09	287.80 ± 14.02 *
**Sigmoidal *E*_max_ model**		
*E*_max_ (%h)	252.21 ± 27.27	1486.97 ± 463.58 *
*EC_50_* (μg·h/mL)	5.49 ± 1.58	43.67 ± 21.45 *
Response factor (*γ*)	1.64 ± 0.16	1.25 ± 0.10

Mean residence time (MRT).
